# Marine Vehicle Sensor Network Architecture and Protocol Designs for Ocean Observation

**DOI:** 10.3390/s120100373

**Published:** 2012-01-02

**Authors:** Shaowei Zhang, Jiancheng Yu, Aiqun Zhang, Lei Yang, Yeqiang Shu

**Affiliations:** 1 State Key Laboratory of Robotics, Shenyang Institute of Automation, Chinese Academy of Sciences, 114 Nanta Street, Shenyang 110016, China; E-Mail: zswsia@126.com; 2 Graduate School of Chinese Academy of Sciences, Beijing 100049, China; 3 LTO, The South China Sea Institute of Oceanology, Chinese Academic of Science, 164 Xingangxi Road, Guangzhou 510301, China; E-Mails: leiyang@scsio.ac.cn (L.Y.); shuyeq@scsio.ac.cn (Y.S.)

**Keywords:** marine vehicles, observing system, sensor networks, path planning

## Abstract

The micro-scale and meso-scale ocean dynamic processes which are nonlinear and have large variability, have a significant impact on the fisheries, natural resources, and marine climatology. A rapid, refined and sophisticated observation system is therefore needed in marine scientific research. The maneuverability and controllability of mobile sensor platforms make them a preferred choice to establish ocean observing networks, compared to the static sensor observing platform. In this study, marine vehicles are utilized as the nodes of mobile sensor networks for coverage sampling of a regional ocean area and ocean feature tracking. A synoptic analysis about marine vehicle dynamic control, multi vehicles mission assignment and path planning methods, and ocean feature tracking and observing techniques is given. Combined with the observation plan in the South China Sea, we provide an overview of the mobile sensor networks established with marine vehicles, and the corresponding simulation results.

## 1. Introduction

Ocean phenomena such as front, wind-driven red tides, and mixing upwelling are rapidly dynamic processes with high spatial and temporal characteristic, which are difficult to observe using regular static mooring observation systems. In order to provide coverage sampling in a regional area and ocean feature tracking in a mobile form, the use of mobile sensor platforms is preferred. Then, it becomes necessary to study the cooperation and optimization between the control and trajectory planning of mobile sensor networks and data assimilation in an ocean dynamic model.

Researchers have carried out a number of ocean observation and environment monitoring programs. Data obtained from these programs has been applied to validate a variety of advanced instruments and ocean sampling strategies. These programs have also provided lots of data for research on ocean physics, biology, chemistry and others. Three large scale sea trials in 2000, 2003 and 2006 had been carried out [1,2] in the Autonomous Ocean Sampling Networks (AOSN) program. A variety of advanced, applicable instruments and regional observing technologies are proposed in ocean observation systems. A group of gliders were utilized to establish a virtual moored array to analyze meso-scale variability and phytoplankton in the Philippine Sea east of Luzon Strait for about 10 days of continuous observation [3]. The European Gliding Observatories (EGO) initiative [4,5] is a gathering of several European countries and oceanographic researchers, interested in ocean observation with underwater gliders. Surface vehicles and underwater vehicles together are exploited to establish a multi-layer model simulation and data assimilation system to collect temperature, salinity and ocean current data for oceanographic feature exploration [6]. The development of *in situ* observing instruments and mobile platforms favors the improvement of ocean observing systemc [7]. Underwater observing sensor platforms mainly include Array for Real-time Geostrophic Oceanography (ARGO) profile floats, fixed underwater observation networks, shipboard oceanographic survey systems, and marine vehicles including Autonomous Underwater Vehicles (AUVs), Autonomous Surface Vehicles (ASVs) and Autonomous Underwater Gliders (AUGs). Researchers prefer to select marine vehicles as sensor platforms carrying platforms for their superior maneuverability and low cost. Marine vehicles are intelligent, mobile, controllable sensor platforms and make possible rapid and adaptive ocean dynamics process observation, especially for near-real-time (NRT) observations of ocean micro-scale phenomena [8,9].

The development of computers and data assimilation techniques provides convenience for prediction and estimation of ocean dynamic process. Ocean models such as Modular Ocean Model (MOM), Princeton Ocean Model (POM), and Regional Ocean Modeling System (ROMS) [10] have developed to a mature status, and become an essential tool to simulate ocean environments. Ocean models are utilized to establish the communication between observations and ocean physical processes. Data assimilation is a technique that melds observation data, and ocean dynamic process together to make efficient, accurate and realistic estimations. Data assimilation methods can be grouped into two categories: sequential assimilation and non-sequential assimilation [11]. The former include optimal interpolation (OI), three-dimensional variation (3DVAR) [12], Kalman Filter, Extended Kalman Filter (EKF), and Ensemble Kalman filter (EnKF) [13]. The latter includes four-dimensional variation (4DVAR) [14] and Kalman Filter Smoother.

According to the observation mission and optimal criteria, the NRT path planning of marine vehicles is closely related to the vehicles’ maneuverability, data estimation and prediction, and cooperative control methods. In view of artificial potentials and virtual bodies, Fiorelli *et al.* [15] proposed a formation control method to detect and track ocean features, such as ocean fronts, upwelling, gradient fields, and eddies. Zhang *et al.* [16] introduced a cooperative KF method to track ocean features in a noisy and changing scalar field, which connects the formation control, data estimation, and the formation optimization together. Jin *et al.* [17] and Susca *et al.* [18] consider the feature boundary as a hidden Markov model (HMM) with separated sample data information collected from multi vehicle platforms, and develop a Page’s cumulative sum algorithm for each vehicle’s boundary track, and estimate the boundary with the data collected from vehicles, the prior knowledge and boundary model. Smith *et al.* [19] designed a mutual observation method: gliders’ trajectory is predicted with the output information from an ocean model, and gliders provide NRT measurement information that is sent back to the ocean model. For the sample of physical and physical biogeochemical dynamics, Lermusiaux [20] combines the ocean model with sample data via data assimilation, and develops a novel adaptive modeling approach with simplified maximum likelihood principles. Leonard *et al.* [21] proposed Objective Analysis (OA) as the evaluation criteria, and then constrained the mobile vehicle-sensor nodes on the parameterized trajectory to execute samples. The desired observation data can be obtained with the optimization of trajectory parameters and number of vehicle sensor nodes. Alvarez *et al.* [8] described networks with drifting profiling floats and gliders for adaptive ocean sampling, and proposed the genetic algorithm to optimize the gliders’ trajectory. Considering the situation where ocean current is comparable to vehicles’ velocity, Davis *et al.* [22] proposed a method to compute an optimal transit route to rapidly reach a specified waypoint based on the ray equations for non dispersive wave propagation. Yilmaz *et al.* [23] applied a mixed integer linear programming (MILP) method to find the vehicles’ path, that where the sample process can maximize the uncertainty of scalar field estimates. Heaney *et al.* [24] proposed a genetic algorithm to assimilate the uncertain regional measurements into the dynamical ocean model.

In this study, a framework of ocean phenomena observing systems with underwater mobile sensor networks is given. The main idea is to integrate the control and plan of the sensor platforms with data assimilation to obtain more sample data. A brief overview of observation system configurations is given in Section 2. Section 3 presents the decomposition and assignment of observation missions. With respect to different kinds of observation missions, the advantage and characteristics of cooperative observations with different marine vehicles are analyzed. Autonomous trajectory planning and optimization of underwater vehicles, mainly for ocean feature tracking and coverage sample of ocean areas, is described in Section 4. Section 5 details the ocean data assimilation and ocean model simulation. Descriptions of semi-physical simulation platforms of marine vehicles are given in Section 6. Finally, Section 7 contains the corresponding simulation results, including the isothermal line tracking in the South China Sea (SCS) and underwater glider dynamic experiments.

## 2. Overall Observing System Configuration

**Figure 1.**
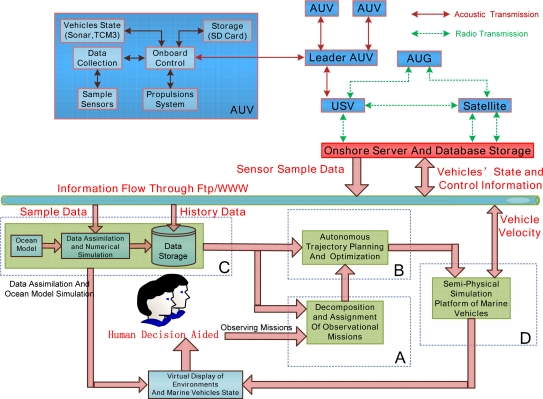
System configuration.

The overall integration of ocean phenomena observation systems includes the following parts (Figure 1): marine vehicles’ onboard system (MVOS), onshore server and database storage system (OSDSS) and data processing and vehicle path planning system (DPVPPS). MVOS includes sample sensors (Conductivity Temperature Depth profiler (CTD)), vehicle sensors (sonar, 3-Axis Compass (TCM3)), propulsion system, storage to store sample information and vehicle dynamic state, and the onboard control part. Onboard control as the management center of the onboard systems collects sample data from CTD; on the other hand, this part controls the vehicle state according to commands from the leader AUV. The onboard control part establishes the connection with other vehicles (or the leader vehicle). Vehicles’ sample information and dynamic information are stored temporarily on the SD card. Multi AUVs are operated in the pre-planning mode, or transmit information to each other through acoustic. Multiple AUVs can follow the leader AUV or work together to complete the observation mission. USV executes ocean surface observations and transfers information between AUG, AUV and OSDSS. AUG can connect and transmit data to OSDSS through satellites or temporarily store sample data on the USV. OSDSS is the center for data storage and data-sharing. Through OSDSS, we can downloaded the control command and trajectory from the vehicles path planning system, and then transmit to each marine vehicle through USV or satellites; on the other side, we can upload and storage the sample data and vehicle’s motion state from the marine vehicles. The internet (WWW/FTP) is selected to connect DPVPPS and OSDSS.

The design of MVOS varies with marine vehicles. In this paper, DPVPPS is described in detail. DPVPPS mainly includes the following modules: (A) decomposition and assignment of observation missions; (B) autonomous trajectory planning and optimization; (C) ocean sample data assimilation and ocean model simulation; (D) semi-physical simulation platforms of marine vehicles. For a specific observation mission, we first decompose the mission and assign the mission to suitable vehicles; then choose favorable history data information from ocean data assimilation module to initialize the mission. Considering the sample data information, optimal criterion and vehicles’ motion characteristics, vehicles’ trajectories are then planned and optimized. After getting new sampling data, we fuse the sampled data and historic data to construct a NRT sample system and provide information for virtual display. The new sample data, data assimilation results and vehicles’ present state are used to predict vehicle trajectory in the next steps. The continuous trajectory is discretized to waypoint form, and then the control law between each two waypoints is designed in marine vehicles’ semi-physical simulation platforms. By using the virtual display, we can obtain general information, and monitor each module’s status and provide assistance to each module in case of emergency.

## 3. Decomposition and Assignment of Observation Missions

Observation and sampling density depends on the rate of change of ocean phenomena. These variations impose various requirements for marine vehicles such as motion mode, communication mode, and endurance. In this study, observations of meso-scale and micro-scales dynamic processes with scale less than 100 × 100 km^2^ is discussed. Typical objectives of an ocean observation mission may include physical oceanography, biological, and ecology. Observation missions can be decomposed into coverage observation in a regional area, ocean feature tracking, emergency response observations, vertical profile observations, and hierarchical observations. Suitable vehicles should be chosen for a particular observation mission. As a result, the basic issues are mission decomposition and marine vehicle selection.

### 3.1. Motion Characteristic of Marine Vehicles

**Figure 2.**
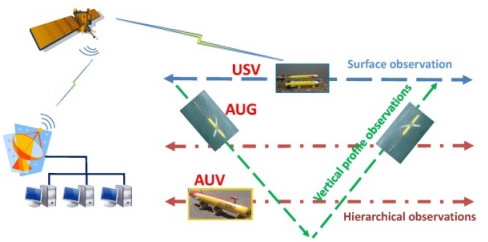
Motion characteristic of marine vehicles.

The characteristics, including communication capability, vehicle endurance, and propulsion system are different for USVs, AUVs, and AUGs. USVs having communications capabilities are close to land robot vehicles can connect to the onshore center in real time with high speed. The endurance of USVs depends on the propulsion systems and energy supply. AUVs communicate with each other with acoustic signals, or cooperate in a pre-planned form. AUVs have a relatively low endurance but good controllability and maneuverability. As a new kind of AUV vehicle, AUGs can take advantage of the hydrodynamic force (lift force) from the main wing and net buoyancy adjustment mechanism to glide up and down, so AUGs have long endurance, but low velocity and maneuverability. AUGs connect with the control center through a satellite when it floats to the sea surface. We select the shore-based centralized control center in planning and optimizing marine vehicles’ trajectory, and transmit the control commands to the vehicles through Iridium or communications transmitters. With the analysis of the marine vehicles’ motion property, three kinds of typical observing missions are defined as follows (Figure 2):

- USV surface observation: These vehicles are suitable for high-precision observations on the sea surface. USVs can be applied for rapid response, emergency management. For example, if satellite communication is lost between a USV and an onshore server, the USV can temporarily store sample data and transfer the information of AUVs and AUGs.
- AUV hierarchical observations: These vehicles are suitable for high-precision observation of different water layers under the sea surface, thanks to their good controllability and maneuverability. AUVs are also preferred for regional observations such as eddies or emergency observations, extreme weather conditions, storm surges, *etc*.
- AUG coverage sampling and vertical profile observations: AUGs can be utilized for vertical profile observation due to their vertical gliding up and down motion. On the other side, AUGs can glide for months with low power consumption. Therefore AUGs are suitable for long duration observations.

It is worth mentioning that vehicle selection and observation mission matching cannot be separated absolutely. With the cooperation of different kinds of vehicles, a variety of observation capabilities can be derived. Considering the endurance of AUGs, we focus on AUG coverage sampling and ocean feature tracking observations, and take USVs and AUVs as additional observing vehicles.

### 3.2. Marine Vehicles Operating Modes for Ocean Observations

The observation processes also have their own focus and requirements with respect to different ocean phenomena. For example, using shipboard CTD cross-section observations, temperature and salinity at different levels are collected. Moored subsurface buoys are mainly proposed to establish *in situ* and long-term observing systems for ocean mixed layers and air-sea interface observations. Drifting buoys can provide ocean temperature data covering a large range of ocean, therefore drifting buoys are chosen to establish the global observation array for ocean forecasting and climate studies. Here, the observing requirements and operating mode for marine vehicles are defined as follows:

- Collaborative observations in a hierarchical plane: We constrain marine vehicles’ path on a parameterized geometric curve, and maintain vehicles’ positions and velocities with certain relationships, then execute coverage sampling observations. In this mode, the three dimensional observations are simplified to two dimensional observations in a horizontal plane at certain depths. This flexible observation mode makes possible quick adjustment of geometric parameters and collaborative relationships, so the sample regional area can be zoomed to maximize the validity of the sample data. This operating mode is suitable for AUVs, USVs, and AUGs. We can get real-time connection or NRT connections from AUVs and USVs, and then design the collaborative control methods. For AUGs, when they float to the sea surface, the shore-based control center gets their position and then a control law is designed to pre-plan the gliders’ distribution during the next diving period.
- Cross section (or profile) continuous observations: AUGs can execute repeat and continuous observations on the selected cross section. Compared to the shipboard CTD observations and ARGO floats, AUGs provide sample data with long duration and high sampling density.
- Virtual mooring array observations: The marine vehicles are controlled in a confined region as an array to execute repeated sampling. This kind of observation is similar to the continuous vertical profile observations achievable with a fixed-point mooring subsurface buoy. A mobile virtual mooring array is made up of multiple marine vehicles. Then the surface observation and cross section observation can be combined together. In addition, through the vehicles’ position control, the suitable anchor point is selected as the virtual anchor array center. This mode is suitable for AUGs.

## 4. Autonomous Trajectory Planning and Optimization

In this section, the trajectory planning and optimization methods for coverage sample observations and ocean feature tracking are discussed. Coverage sample observation is an observation task with multiple marine vehicles working together to sample an ocean regional area uniformly. From the coverage sample, rough information observations can be obtained. After that, we focus on an ocean feature of interest, such as upwelling, or ocean front, and propose multiple vehicles combined with the NRT observation data to track the origin and boundaries of the feature.

### 4.1. Coverage Sample Observations

**Figure 3.**
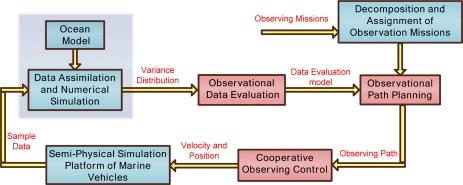
Coverage sample observation.

The system frame of coverage sample observation is shown in Figure 3. The sample data obtained from the marine vehicles are assimilated in the ocean model, and then the sample data variance distribution is evaluated to obtain prediction information of the observation objective. The data evaluation criterion decides the vehicles’ path planning and sample objective. Through mission assignment, we choose the suitable vehicle, and integrate variance distribution and prediction of sample data to plan the marine vehicles’ paths.

It is difficult to get comprehensive information with a single vehicle, so multiple vehicles are required to sample in a formation or maintain some relative position with respect to each other to obtain multiple data sets from different locations at the same time. KF and EKF are utilized for the sample instrument noise and the ocean spatially correlated noise. The ocean features are nonlinear, and cannot be expressed in explicit functions. However, with the understanding and the knowledge of the ocean features, EKF provides an approximate method to describe and estimate their variance. The sampling processes are affected by the ocean currents, marine vehicles’ velocity and endurance, so the cooperative observation control module optimizes vehicles’ formation and plans vehicles’ desired velocity to adjust for any disturbance of the environment. Lastly, marine vehicle platforms execute the desired velocity control and data sampling. Detailed descriptions of observation data evaluation, observation path planning and cooperative observation control are as follows:

- Observation data evaluation: Criteria of observation data evaluation determine the effectiveness of data sampling. From numerical simulation and data assimilation, ocean environment prediction results can be achieved. In this study, the criterion is defined as the output uncertainty variance of the environmental prediction. The environmental prediction can be obtained from ocean numerical simulation in a constant-interval sequence form. It is known that, the longer of interval, the less the accuracy of the prediction results. Distribution of uncertainty variance reflects the accuracy of the prediction results: uncertainty variance of adjacent sequence reflects the spatial correlation of the ocean dynamic process, and uncertainty variance of the same network cell at different times reflects the temporal correlation of ocean dynamic process.
- Observing path planning: Combining objectives, the ability of marine vehicles and environmental constraints, we transform the observation path and constraints as objective functions and constraint functions. The objective functions can be the shortest time, minimum energy, or validity of the observation data. In this study, the distribution of uncertainty prediction variance is selected as the objective function to improve the accuracy of environmental predictions. Repeated observations are carried out at the position of maximum uncertainty variance to access the interesting data. The constraints include velocity and endurance of marine vehicles, initial sampling position constraints, and observation operation (trajectory) mode, environmental constraints and so on. Mixed Integer Linear Programming (MILP) is proposed to solve the path optimal problems and design suitable marine vehicles’ trajectories to obtain comprehensive and uniformly distributed sample data. For continuous cross section observation, vehicles’ trajectory is restricted to a straight line in a longitudinal plane. In virtual observation mooring array observation, marine vehicles are constrained in a small radius circle, and then the trajectory of the array circle center is planned. For hierarchical observations, the vehicles’ trajectory is pre-planned on the closed-loop curve, such as a circle, ellipse, or square. The path planning is simplified to optimize the close-loop curve parameters (such as the center position of the curve, area covered by the curve, *etc.*), the number and location position of the vehicles. Ocean currents affect the vehicles’ motion and dynamics, especially AUGs which operate at a low velocity compared to AUVs and USVs. Current data is transformed into a three dimensional grid data and compensated using the vehicles’ velocity.
- Cooperative observation control: During observation processes, marine vehicles should operate collaboratively and maintain their relative positions in a formation. The cooperative control includes two types named “loose collaboration” and “close collaboration”. For the “loose collaboration”, we mainly plan each single vehicle’s path and control the vehicle’s dynamic model to maintain its observation path. For example, multi-profile continuous observation and virtual mooring observations are “loose collaboration” observations. In the “close collaboration” mode, multiple vehicles are required to have strict distribution to maintain their relative positions. For example, multiple vehicles cooperatively sampling on a closed curve is a “close collaboration”. In this situation, we design the control law to maintain the desired interval by measuring the curve arc length between adjacent vehicles. The vehicles’ dynamic control and the cooperative trajectory control law are designed separately. The continuous trajectory is discretized into waypoints, and the vehicles’ dynamic control law drives each vehicle to reach the next waypoint in the shortest time. A particle agent model is used to simplify the cooperative trajectory control design. In deep sea, two adjacent AUG exit points are about 5–7 km apart, and time interval is about 5 ∼ 7 hours, so the cooperative control method for AUGs has to magnify many times in the spatial and temporal aspect, compared to land robots. It is possible that sampling data from different AUGs is not synchronized, since AUGs may not float to the sea surface at the same time. In this situation, the gliding velocity and position are pre-planned from data assimilation results.

### 4.2. Ocean Feature Tracking

**Figure 4.**
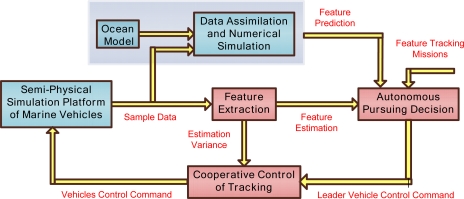
Ocean feature tracking.

After the rough information of the regional area is obtained, multiple vehicles are introduced to track the feature(s) of interest in the area. Figure 4 shows the frame and processes of ocean feature tracking.

First, the phenomena and variations are described as a mathematical expression by which the vehicles can track and observe in feature extraction module. For example, to track temperature upwelling, the temperature is described as a scalar field with the maximum (or minimum) as the center and gradient as the variation direction and magnitude of upwelling. In order to avoid the limitations of observation information, multiple vehicles are proposed to move in a formation or rotational formation to obtain comprehensive information of the scalar field, and multiple sample data are employed to estimate the feature variation. The direction of movement of multiple vehicles depends on the feature extraction results and tracking missions. After the movement direction is certain, a leader-follower form is applied, that the leader vehicle tracks the movement direction, and multiple vehicles follow the leader vehicle to achieve the sample data. Those movements are defined as autonomous pursuing decision and multi vehicles cooperative control of tracking. There are certain noises in the observation data, caused by the complex background field noise and instrument noise, so KF, EKF are introduced in vehicles’ cooperative control module to reduce the observation errors caused by instruments and temporal-spatial variations. Detailed descriptions are as follows:

- Feature extraction: Typically extracted ocean features include upwelling, fronts, gravity waves, tides, eddies. After the rough information of the ocean feature is received, an appropriate number of vehicles is selected to carry out the tracking missions. For example, when we track the two-dimensional temperature field T, the mission is described as tracking of T, ∇T_x_, ∇T_y_, where [∇T_x_, ∇T_y_] is a gradient that reflects the variation of temperature. Three vehicles are enough to complete the mission if the impact of higher order derivatives of the scalar field T is neglected. Then multiple vehicles are driven to move forward intelligently along the gradient directions or in an anti-gradient direction, or an orthogonal direction to the gradient, which depends on the observation objective.
- Autonomous pursuing decision: Autonomous pursuing decision reflects multi vehicles’ cluster movement direction. The leader (or virtual leader) vehicle is defined as the multiple vehicles’ formation center, and the control law of the leader’s velocity and steering velocity is designed to track the feature. Then other vehicles are controlled to follow the leader in a formation. Autonomous pursuing decision combines the virtual leader vehicle tracking strategy and the actual track goals together, for example, when tracking temperature contours, first the pursuing strategy is designed to control the vehicle to move close to the contours, and then move along the temperature contours. In this process, many constraints and limitations should be taken into account, such as the actual vehicles’ speed limitation, the error between the movement direction and expected direction. If the feature is lost, the predictive information can be adopted to re-position.
- Cooperative control of tracking: After the decision of the leader moving direction is determined, the control law is applied (including velocity and location) to drive multiple vehicles following the leader, and maintain a desired formation. Multiple vehicles’ formation control includes rotation, scaling and translation terms. The scaling of formation can change the size of the observation area. The qualities of observation data are different with respect to the sample location. For example, the gradient estimation depends on sample data T and distance between vehicles (P_i_, i=1,2,3) and vehicle leader P_C_ as Equation (1) shows:
  (1) $$T=T_{i}+\left[{\nabla T}_{x}{\nabla T}_{y}\right]{\left[P_{i}-P_{C}\right]}^{\prime },i=1,2,3$$

So in order to enhance the validity of sample data, the distance between vehicles as in Equation (1) are changed periodically to scale and rotate the vehicles’ formation. As a result, the sample data estimation and formation design can be combined together to get rich sample data that reflects the feature variation and characteristics.

## 5. Ocean Data Assimilation and Ocean Model Simulation

**Figure 5.**
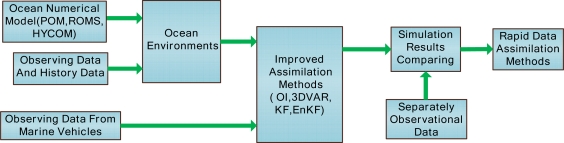
Ocean model and data assimilation.

Ocean models and historical sample data from satellites and *in situ* observations provide convenience for the construction of ocean environment models. Based on the knowledge of ocean environments and sample data from vehicles, many kinds of methods such as optimal interpolation, variation method and EnKF are utilized in data assimilation. We compare the assimilation results, and select the rapid data assimilation method for near real time observations. Figure 5 gives the processing flow scheme of data assimilation. There are appropriate and mature ocean circulation models (POM, ROMS, HYCOM) for ocean observations. In order to progressively enhance the temporal and spatial resolution of ocean simulation results, the sample data of observing region is refined level by level from 15′ × 15′, to 5′ × 5′, and then 2′ × 2′. The low resolution simulation results provide the initial conditions and boundaries for the high resolution of ocean model simulations. Numerical simulations of regional observations include the following two steps: first, the ocean model starts up with the forcing field of climatology. When the ocean model becomes stable, we can get the climatological annual cycle. With these basic analyses, the driven factors, such as wind, sea surface heat flux, and freshwater flux, are added to get a NRT ocean environment simulation. Numerical modeling and simulation of regional ocean areas can provide ocean dynamic simulation environments, which are useful for verification of the correctness and effectiveness of the relevant sample data. On the other side, the simulation results can be applied to construct the background for the data assimilation.

With the help of historical sample data and numerical model simulation results (for ocean regional areas that lack observation data), we select a suitable observation mode, and different kinds of marine vehicles to sample the relative region, and then construct the ocean feature environment model. The sample data collected by vehicles are the original data, and can be fused with the historical data for ocean background construction. The data assimilation results are evaluated on various aspects, such as demographic characteristics of the sample data, mean of the sample and RMS (root mean square) errors. The core concept of data assimilation is the definition of error and error estimation, including the background error (ocean dynamic model error) and observation error. The introduction of a background error covariance matrix that is suitable for the actual situation is important and difficult if there is less background information. Here, the recursive filter method and sequential three-dimensional variational analysis (Sequential 3DVAR) are utilized to construct the background error covariance matrix. With the consideration of the effectiveness and efficiency of data assimilation methods, the rapid data assimilation methods, that suitable for the marine vehicles’ observation system are ultimately determined.

## 6. Semi-Physical Marine Vehicle Simulation Platforms

**Figure 6.**
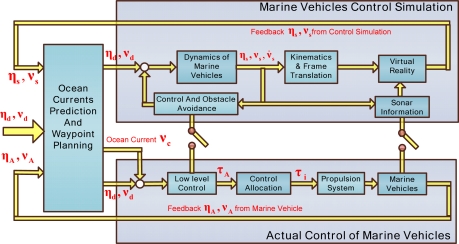
Semi-physical simulation platforms.

The observation sensor nodes are mainly composing of AUVs, AUGs, ASVs, and the dynamics and motion control of marine vehicles also have a significant impact on the sensor networks. This section gives a general control frame for marine vehicles as the following three parts (Figure 6): marine vehicle control simulation (MVCS), actual control of marine vehicle (ACMV), and ocean currents prediction and waypoint planning (OCPWP). Ocean currents have a significant influence on the vehicles’ motion, especially for AUGs. Sometimes, ocean currents are considerable compared to an AUG’s velocity, or even bigger than an AUG’s velocity. Ocean currents are estimated and then compensated with the vehicles’ velocity. As we know, due to the ocean currents and other external disturbances, the feedback velocity, position from MVCS and ACMV are different. From the multiple vehicles trajectory plan, we obtain the desired velocity ν_d_ and position η_d_ in the static water without consideration of ocean currents, so the vehicles control velocity v_A_ for ACMV may be different from v_d_. From the difference between actual control feedback information ν_A_, η_A_ and simulation feedback information ν_s_, η_s_, ocean current ν_c_ can be predicted. Then the desired ν_d_, η_d_ and ocean currents ν_c_ are added to ACMV, and the desired ν_d_, η_d_ are utilized in MVCS. Through the simulation and actual control module, current predictions are updated cyclically from ν_A_, η_A_ and ν_s_, η_s_. Current prediction information from the previous cycle is applied to correct the vehicle’s present velocity. If the vehicle navigated the downstream, we have v_A_ < v_d_, otherwise, we have v_A_ > v_d_.

From the nominal vehicle dynamic model, MVCS provides an intuitive impression about the vehicle motion in the virtual reality, and provides a convenient condition for handing any emergency, collision avoidance, ocean current compensation, and debugging the advance control methods. Due to some velocity V, and position η from OCPWP, the simulation results can be achieved from the nominal dynamic model. Then through the coordinate frame transforms, the marine vehicles’ velocity, trajectory and position are transformed to the inertial frame, and finally, displayed on the virtual reality screen. ASVs can easily communicate with the shored control center in real-time mode, so the simulation component and actual control component can feedback information online from each other. AUVs and AUGs usually work in a pre-planning mode; therefore, it is convenient to switch the information transformation from real-time contact mode to the pre-planning mode or NRT mode.

The dynamic models of AUV, AUG and ASV have a general form as in Equation (2) with subtle differences caused the propulsion systems used [25]:

(2) $$M\dot{\nu }+D\left(\nu \right)\nu +C\left(\nu \right)\nu +g\left(\eta \right)=\tau$$

where ν, η, τ are the velocities, position, and propulsion force, respectively; M, D(ν), C(ν), g(η) are the inertial mass matrix, hydrodynamic damping matrix, rigid body coriolis and centripetal matrix, and restoring forces and moments. AUVs and ASVs are propelled by thrusters, fins and rudders, so many advanced control methods are used in these vehicles’ control. AUGs are propelled by net buoyancy and wings’ lift force. As a result, AUGs’ maneuverability is poor. On the other side, AUGs usually glide in steady states, and the switch time from initial state to a steady gliding process is short compared to the steady gliding process, so it is necessary to modify and correct the nominal dynamic model with lots of AUG ocean and pool experiments. Lastly, the relationships between gliding state and net buoyancy, position adjustment of inner movable mass block, can be applied for AUG path planning and optimization.

ACMV mainly includes the low-level control, control allocation, and propulsion systems. We get the control force from the low-level control and then assign it to each thruster and rudder through control allocation. For AUGs, the control allocation is reflected as the adjustment of net buoyancy, the position of an inner movable mass block. Then we get ν_A_, η_A_ from vehicle dynamic sensors and feedback to the ocean currents prediction and waypoint planning module.

## 7. Simulation Results

In this section, partial simulation results of the observation system are given. Firstly, the isothermal line tracking in a temperature scalar field is given as an example of ocean feature tracking, and secondly, experimental results of AUGs are described as an example of semi-physical marine vehicle simulation platforms.

### 7.1. Feature Tracking with the Archived Data in the SCS

**Figure 7.**
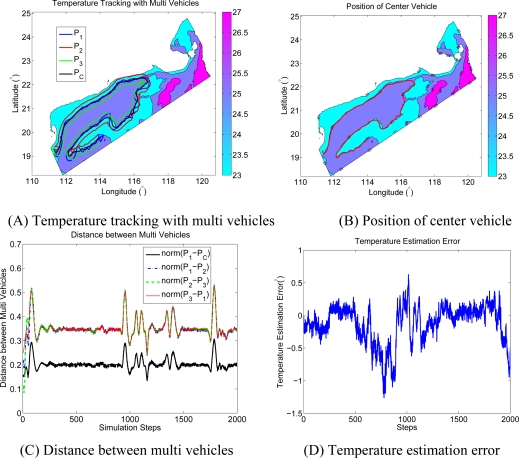
Temperature isothermal line tracking with marine vehicles.

The CTD data from the Northern SCS coastal Oceanographic Process Experiment Pilot-Project (SCOPEPP) cruise from 30 June to 13 July 2008 are used in the simulation. The sample data characterizes the temperature and salinity profile along the transactions during the cruise. We only focus on the isothermal line tracking at 35 m below surface. Since resolution of the sample data is low, a longitude is identified as a unit length, and three vehicles are employed to track the temperature isothermal line of 25°. The multiple vehicles’ formation is an equilateral triangle (ΔP_1_P_2_P_3_), and P_C_ is the virtual leader vehicle in the formation center. The distance between each vehicle and the equilateral triangle center is 0.2 unit length. The trajectories of each vehicle (P_1_, P_2_, P_3_, and P_C_) are shown in Figure 7(A). The connecting line of vehicle 1(P_1_) and the virtual vehicle center (P_C_) coincides with the isothermal line, and the connecting line of vehicle 1 (P_1_) and vehicle 3 (P_3_) is orthogonal to the isothermal line. The rotational angular velocity of the formation depends on the curvature of the ocean feature, and the trajectory path length of the vehicle is different from each other. Figure 7(A) shows that, the path length of vehicle 2 is bigger than that of vehicle 3, as the trajectory of vehicle 2 is at the outside of the isothermal line most of the time. The path length of vehicle 1 is almost the same as that of the center vehicle, for its trajectory coincides with that of the center vehicle. As a result, the endurance and velocity of the vehicle should be seriously considered and selected. A Kalman Filter is utilized to estimate the temperature at the virtual vehicle center (P_C_) with the temperature information from P_1_, P_2_, and P_3_. Figure 7(C) gives the distance variations between the multiple vehicles. The convergence of the multiple vehicles’ formation is better in the field where the temperature changes are moderate. The isothermal line has great bending in the scalar field where the temperature changes rapidly, so when vehicles pass through these areas, the path length of each vehicle has a large difference; and formation is adjusted automatically according to the sample information from P_1_, P_2_ and P_3_. As a result, vehicle formation in these areas has small oscillations. Trajectory of the virtual vehicle center is shown in Figure 7(B). Temperature estimation error between the true value in P_C_ and the estimation result from P_1_, P_2_, and P_3_ is shown in Figure 7(D). From Figure 7(B,D), it can be seen that, the multiple vehicles’ formation center is around the 25° isothermal line with a small estimation error.

### 7.2. Dynamic Control and Simulation of Marine Vehicles

AUG is selected as a special example to discuss the vehicles dynamic and control, for AUG’s maneuverability is low compared to other kinds of vehicles. The glider prototype was designed from 2003, and the engineering versions were completed in 2007. The glider of engineering versions can dive down to 800 meters depth, with durance of 500 kilometers. The dynamic model of AUG that will be utilized in the observing systems has been discussed in [26] as:

(3) $$\begin{array}{l} \dot{\nu }=M^{-1}\left\{-\dot{M}\nu +\left[\begin{array}{c} P\times \Omega \\ \Pi \times \Omega +P\times V \end{array}\right]+\left[\begin{array}{c} m_{b}g\left(R_{\text{EB}}^{T}k\right)\\ \left(m_{\text{mr}}r_{\text{mr}}+m_{\text{rb}}r_{\text{rb}}+m_{b}r_{b}\right)\times (R_{\text{EB}}^{T}k) \end{array}\right]+\left[\begin{array}{c} F\\ T \end{array}\right]\right\}\\ {\dot{m}}_{b}=u_{b} \end{array}$$

Glider motions mainly include steady vertical motion and three dimensional spiraling gliding motions. With the assumption that the position of m_mr_ is fixed at r_mr_, and with a constant net buoyancy which implies that the control input ṁ_b_ = 0, glider’s equilibrium equations in three dimension are:

(4) $$\{\begin{array}{c} P\times \Omega +m_{b}g\left(R_{\text{EB}}^{T}k\right)+F=0\\ \Pi \times \Omega +P\times V+\left(m_{\text{mr}}r_{\text{mr}}+m_{\text{rb}}r_{\text{rb}}+m_{b}r_{b}\right)\times \left(R_{\text{EB}}^{T}k\right)+T=0 \end{array}$$

When a glider glides in the spiraling motion with a constant velocity, the pitch and roll angles are constant [27]. This situation implies that $R_{\text{EB}}^{T}k$ is constant. By taking the derivative of $R_{\text{EB}}^{T}k$ with respect to time [28], we get:

(5) $$\Omega \times \left(R_{\text{EB}}^{T}k\right)=0$$

**Figure 8.**
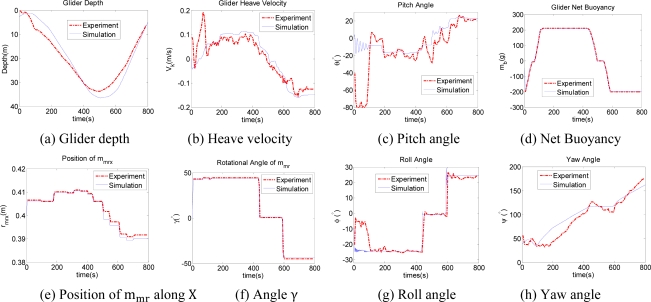
Underwater glider simulation and experiments.

From Equation (5), it is known that the underwater glider glides with constant angular velocities along a circular helix which is aligned with the direction of gravity, and the angular velocity is $\Omega =R_{\text{EB}}^{T}{k\omega }_{3}$. We select the net buoyancy mass m_b_, position r_mrx_ and rotational angle γ of r_mr_ as the control input parameters, and give a description of a glider experiment performed during May 2011 in Songhua Pool, Jilin Province, China. CTD is used to get glider depth information and the digital compass module TCM3 (3-Axis Compass) to get glider attitude angles. Due to the depth limitation of the pool, the glider gliding depth is restricted within approximately 40 m. The sampling periods of TCM3 and CTD are approximately 6 seconds. In the experiment, the position and rotational angle (pitch angle θ, roll angle ϕ) of m_mr_ are adjusted intermittently with TCM3 feedback information, and in a Matlab simulation, the open loop control is utilized to verify the glider’s hydrodynamic properties. The equilibrium point of buoyancy and position of mass m_mr_ are m_b_ = 0 and r_mrx_ = 0.4016 m. In the experiment, the gliding control mission in one gliding period is given as buoyancy m_b_ = 0.2 kg, and offset of the linearly and rotationally movable mass as ||Δ r_mrx_ || = 0.01 m and pitch angle ||θ|| = 20°. Figure 8(a) shows glider depth information in the vertical plane. Considering the limitation of velocity sensor, we differentiate depth with respect to time to get the velocity along k, which is shown in Figure 8(b). The glider pitch angle is approximately 20°, as shown in Figure 8(c). Figure 8(d–f) give glider control inputs of m_b_, r_mrx_ and γ. || Δ r_mrx_ || which are 10 mm approximately and the net buoyancy is 0.2 kg, and rotational angle γ is 48°. Figures 8(g,h) show roll angle and yaw angle with respect to the variation of γ. Figure 8(h) shows that the turning direction is the same with opposite input of γ during turning up and down. Both the input γ and direction of glider lift force change oppositely during up and down. The centripetal force is a projection of lift force with a sine function of γ. As a result, the turning direction is the same. The simulation results are agreement with the experiment in most cases, with a little difference caused by the flow, the glider mass distribution, and other disturbances. The comparison shows that the hydrodynamic coefficients and dynamic model predict the overall glider dynamic accurately.

## 8. Conclusions and Future Works

In this paper, a general introduction to architecture and protocol design for underwater mobile sensor networks is given. The main structure and methods of the observation system have been discussed, including mission assignment, marine vehicle dynamic control, numerical simulation of ocean models and data assimilation, and autonomous trajectory planning and optimization. Partial simulation results using cruise data in the SCS have been given to demonstrate the effectiveness of the system. In the future, more deep sea experiments for AUG will be utilized to modify the dynamic model, and enhance the glider’s performance. Sea currents from advanced Doppler current profilers (ADCPs) and other observation instruments will be integrated into the semi-physical simulation platforms for current estimations. The cooperative working of AUGs, USVs, and AUVs will be thoroughly considered for the information transmission and control delay. The dynamic analysis will be integrated to the path plan and formation control, so that, the limitations and constraints between these two parts can be considered. The limitations of velocity, time delay, and endurance in multiple vehicle formation control will be analyzed to establish a cooperative control system. The ocean model and data assimilation methods will be applied in SCS to mainly analyze the temperature and salinity variation. These parts will be integrated and fused into an overall system. The observation system will also be verified with more observation experiments in the SCS.
